# Safety and Outcomes Associated with the Pharmacological Inhibition of the Kinin–Kallikrein System in Severe COVID-19

**DOI:** 10.3390/v13020309

**Published:** 2021-02-16

**Authors:** Eli Mansour, Andre C. Palma, Raisa G. Ulaf, Luciana C. Ribeiro, Ana Flavia Bernardes, Thyago A. Nunes, Marcus V. Agrela, Bruna Bombassaro, Milena Monfort-Pires, Rafael L. Camargo, Eliana P. Araujo, Natalia S. Brunetti, Alessandro S. Farias, Antônio Luís E. Falcão, Thiago Martins Santos, Plinio Trabasso, Rachel P. Dertkigil, Sergio S. Dertkigil, Maria Luiza Moretti, Licio A. Velloso

**Affiliations:** 1 Department of Internal Medicine, School of Medical Sciences, University of Campinas, 13083-887 Campinas, São Paulo, Brazil; eliemansour27@hotmail.com (E.M.); palma.andrecitroni@gmail.com (A.C.P.); haysa_@hotmail.com (R.G.U.); costaribeirolu@gmail.com (L.C.R.); anaflavia.bsousa@gmail.com (A.F.B.); thya_goo@hotmail.com (T.A.N.); marcus.agrela@gmail.com (M.V.A.); santosth@unicamp.br (T.M.S.); trabasso@gmail.com (P.T.); lmoretti@unicamp.br (M.L.M.); 2 Obesity and Comorbidities Research Center, University of Campinas, 13083-864 Campinas, São Paulo, Brazil; brunabombassaro@gmail.com (B.B.); monfortpiresm@gmail.com (M.M.-P.); rlcamargo.bio@gmail.com (R.L.C.); pa.eliana@gmail.com (E.P.A.); 3 School of Nursing, University of Campinas, 13083-887 Campinas, São Paulo, Brazil; 4 Autoimmune Research Lab, Department of Genetics, Microbiology and Immunology, Institute of Biology, University of Campinas, 13083-862 Campinas, São Paulo, Brazil; nbrunetti.bio@gmail.com (N.S.B.); asfarias@unicamp.br (A.S.F.); 5 Department of Surgery, School of Medical Sciences, University of Campinas, 13083-887 Campinas, São Paulo, Brazil; aefalcao@gmail.com; 6 Department of Radiology, School of Medical Sciences, University of Campinas, 13083-887 Campinas, São Paulo, Brazil; rakapolo@yahoo.com (R.P.D.); dertkigil@uol.com.br (S.S.D.)

**Keywords:** angiotensin converting enzyme 2, bradykinin, coronavirus, inflammation, lung

## Abstract

**Background:** Coronavirus disease 19 (COVID-19) can develop into a severe respiratory syndrome that results in up to 40% mortality. Acute lung inflammatory edema is a major pathological finding in autopsies explaining O_2_ diffusion failure and hypoxemia. Only dexamethasone has been shown to reduce mortality in severe cases, further supporting a role for inflammation in disease severity. SARS-CoV-2 enters cells employing angiotensin-converting enzyme 2 (ACE2) as a receptor, which is highly expressed in lung alveolar cells. ACE2 is one of the components of the cellular machinery that inactivates the potent inflammatory agent bradykinin, and SARS-CoV-2 infection could interfere with the catalytic activity of ACE2, leading to the accumulation of bradykinin. **Methods:** In this case control study, we tested two pharmacological inhibitors of the kinin–kallikrein system that are currently approved for the treatment of hereditary angioedema, icatibant, and inhibitor of C1 esterase/kallikrein, in a group of 30 patients with severe COVID-19. **Results:** Neither icatibant nor inhibitor of C1 esterase/kallikrein resulted in changes in time to clinical improvement. However, both compounds were safe and promoted the significant improvement of lung computed tomography scores and increased blood eosinophils, which are indicators of disease recovery. **Conclusions:** In this small cohort, we found evidence for safety and a beneficial role of pharmacological inhibition of the kinin–kallikrein system in two markers that indicate improved disease recovery.

## 1. Background

Acute respiratory failure is the leading cause of death in coronavirus disease 19 (COVID-19) [1,2]. In severe cases, there is a progressive shortening of breath and hypoxemia that often require supplementary oxygen or mechanical ventilation [2,3]. Chest computed tomography (CT) scans reveal multiple lung opacities that are both central and peripheral and can present as ground-glass and consolidation types, usually involving two or more lobes [4,5]. Histological examination of lung autopsy specimens identified angiocentric inflammation with mild/severe interstitial edema, linear intra-alveolar fibrin deposition, and early intra-alveolar organization [6]. These findings provide an anatomopathological basis that could explain O_2_ diffusion failure and hypoxemia, revealing that acute inflammation is crucial for the severe progression of disease [7]. This was further confirmed by the demonstration of reduced mortality in patients with severe COVID-19 who were treated with dexamethasone [8]. Despite intensive efforts to find effective therapeutic strategies, mortality rates remain high, ranging from 5% to 49% in patients admitted to hospitals and intensive care units (ICUs) [9,10,11,12,13].

SARS-CoV-2, the pathogenic agent of COVID-19, enters human cells employing angiotensin-converting enzyme 2 (ACE2) as a receptor [14]. Lung alveolar epithelial cells and macrophages express high levels of ACE2 and thus are primary targets for SARS-CoV-2 [15,16]. At least two studies have shown that, different from the influenza virus, the SARS-CoV-2 infection is accompanied by low expression levels of type I and II interferons and a massive production of inflammatory cytokines [17,18], suggesting that this virus has an efficient immunomodulatory capacity that could explain, at least in part, its pathogenicity [7].

As for other emerging viral diseases, the development of vaccine and effective antiviral agents are expected to provide maximum advance in prevention and therapeutics [19]. However, even in the face of the current global efforts to develop such strategies, years may pass before successful therapies are available for worldwide use [20,21]. Thus, drug repurposing has emerged as a potentially useful strategy to gain time and promote some advance in the battle against SARS-CoV-2 [22]; in this scenario, existing antiviral drugs are undisputedly the natural candidates, and a number have been tested so far [23]. Unfortunately, clinical trials with lopinavir–ritonavir and remdesivir resulted in no change in mortality or severity of disease [11,22], whereas the combination of interferon beta-1b, lopinavir–ritonavir, and ribavirin resulted only in a shorter median time to negative nasopharyngeal swab, suggesting that viral shedding could be abbreviated [24].

As proposed elsewhere, drugs that act in distinct stages of the SARS-CoV-2 cell entrance and replication cycle could promote beneficial outcomes in the treatment of COVID-19 [23]. Due to its action as a SARS-CoV-2 cell entry receptor, ACE2 has emerged as a primary candidate for pharmacological approaches aimed at reducing viral entrance and mitigating disease severity [25,26]. It has been suggested that in addition to mediating viral entrance, ACE2–SARS-CoV-2 physical interaction could modify ACE2 catalytic activity, impacting on the regulation of three independent (but rather integrated) systems—bradykinin, angiotensin, and coagulation [27,28,29,30]. This could explain, at least in part, the development of the most lethal triad in COVID-19 (i.e., severe lung inflammation, acute cardiovascular failure, and thromboembolic events).

Physiologically, ACE2 catalyzes the reaction that inactivates the inflammatory substance DR9-bradykinin [31]. In an experimental model of lung inflammation, the inhibition of ACE2 increased inflammatory cell recruitment and alveolar edema, whereas the inhibition of bradykinin protected the lungs from severe inflammatory damage [31]. Following SARS-CoV-2 binding, ACE2 is internalized to endosomes, resulting in a subcellular shift that could affect its capacity to regulate bradykinin degradation [18,28,32,33,34,35]. Here, we hypothesized that increased bradykinin levels could be one of the mediators of severe lung inflammation in COVID-19, and pharmacological inhibition of bradykinin could beneficially change the course of severe SARS-CoV-2 infection. A similar mechanism has been proposed for SARS-CoV lung damage [36].

In this case-control study, we evaluated 30 patients with severe COVID-19 that were randomized to one of the following treatments: standard care, icatibant (a bradykinin receptor 2 inhibitor) or inhibitor of C1 esterase/kallikrein (iC1e/K). We show that pharmacological inhibition of the kinin–kallikrein system in severe COVID-19 is safe and promoted significant improvement of lung CT scores and increased blood eosinophil counts.

## 2. Methods

### 2.1. Study Design and Patients

This was a case control study conducted at the Clinics Hospital, University of Campinas. Enrollment occurred from 22 April 2020 to 14 June 2020. There was a 1:1:1 allocation ratio to icatibant plus standard care vs. iC1e/K plus standard care vs. standard care alone. The study followed the principles of the Declaration of Helsinki. Inclusion criteria were age at least 18 years at the time of signing the consent form, symptom duration of 12 days or less upon recruitment, SARS-CoV-2 diagnosis by RT-PCR method according to the Berlin–Charité protocol [37], diagnosis of COVID-19 typical pneumonia confirmed by CT of the chest and scored by two lung expert radiologists, SpO_2_ ≤ 94% in ambient air or Pa0_2_/FiO_2_ ≤ 300 mmHg, willingness of study participants to accept randomization to any assigned treatment arm, patient or responsible family member signing the consent form, and agreement that patient should not enroll in any other experimental study prior to completion of the 28-day follow-up. Exclusion criteria were pregnant or breastfeeding women (beta-human chorionic gonadotrophin (HCG) level was determined in all eligible women of childbearing age); severe renal impairment (estimated glomerular filtration rate ≤ 30 mL/min/1.73 m^2^), patients receiving continuous renal replacement therapy (hemodialysis or peritoneal dialysis), or previous renal transplant; severe liver disease (aspartate aminotransferase (AST) or alanine aminotransferase (ALT) 5X above the reference value); HIV infection (HIV was tested for in all eligible patients); or patients with any other immunodeficiencies, previous diagnosis of cancer, previous diagnosis of hereditary angioedema, previous ischemic myocardial disease, previous thromboembolic disease, current use of immunosuppressive drug therapy, or use of any experimental treatment for SARS-CoV-2 infection within 30 days prior to screening. This study was approved by the local Ethical Review Committee of the Clinics Hospital of the University of Campinas (protocol CAEE: 30227920.9.0000.5404), which granted a waiver of consent because treatment concerned a licensed drug that would be given in an off-label setting. Written consent to participate in this study has been obtained from all patients or substitute decisionmakers (for patients lacking decision-making capacity).

### 2.2. Sample Size

Overall, both icatibant and iC1e/K can present up to 55% adverse effects that range from minor skin reactions to liver and cardiac abnormalities. Employing alpha = 0.05 and power = 80%, we determined that at least 9 patients per arm would be sufficient to evaluate safety.

### 2.3. Randomization

The randomization list was generated by an independent researcher with no involvement in the study. A software-based approach (www.sealedenvelope.com, (accessed on 20 April 2020)) was used to generate the allocation sequence. Sealed and sequentially numbered opaque envelopes, containing the allocation arms, were prepared in advance according to the randomization list. The project coordinator and investigators in each unit of the hospital enrolled the participants. Once the eligibility criteria were met, the envelope was opened, and the therapy was prescribed according to the arm specified in the envelope.

### 2.4. Blinding

This was an open-label study; thus, neither the trial participant nor the care providers were blinded. However, data entry staff and the data manager were blinded. Data were sent to the data entry staff by code, and the study arm was never specified. Data entry staff and data managers were not part of the hospital staff and were never given access to any clinical data belonging to the patients. The Data Safety Monitoring Board was unblinded.

### 2.5. Procedures

Group 1, standard care. Group 2, Firazyr^®^ (icatibant acetate 30 mg (3.0 mL of 10 mg/mL solution)) subcutaneous injections in the abdominal area were administered at intervals of 8 h for 4 days plus standard care. Group 3, Berinert^®^ (human plasma-derived C1 esterase/kallikrein Inhibitor) administered at a dose of 20 IU/kg body weight on day 1 shortly after recruitment and on day 4 (each vial contains 500 IU of C1 esterase/kallikrein inhibitor as a lyophilized product for reconstitution with 10 mL of sterile water for injection) plus standard care. Upon medical decision, patients received antibiotics, antithrombotic therapy, oxygen support, non-invasive and invasive mechanical ventilation, vasopressor drugs, stress doses of corticosteroids, and renal support therapy. The criteria for eventual discontinuation of the intervention were withdrawal of consent, grade 4 adverse reaction or allergy to the drug, and protocol violation.

### 2.6. Clinical, Radiological, and Laboratory Monitoring

Diagnosis of SARS-CoV-2 infection was performed by RT-PCR in nasopharyngeal swab samples. Once patients were included in the study, clinical data were obtained continuously and uploaded once a day on the data management system. CT scans were performed at admittance and at days 14 and 28, or otherwise at hospital discharge. CT scan scoring was performed by two experienced radiologists and followed guidelines published elsewhere [37]. For scoring, radiologists looked for the presence of ground glass opacity, crazy-paving pattern, and consolidation. Each of the five lung lobes was scored 0–5 according to the following parameters: 0, no involvement; 1, <5% involvement; 2, 5–25% involvement; 3, 26–49% involvement; 4, 50–75% involvement; 5, >75% involvement. Blood samples were collected on admittance and at days 7, 14, 21, and 28, or otherwise at hospital discharge. Additional clinical, radiological, and laboratory tests were performed at any time at the discretion of the medical staff. Hematological and biochemical parameters were determined in blood samples using automated methods. Cytokines were determined using ELISA kits, according to the recommendations provided by the manufacturers (interleukin-1beta, RD Systems—catalog number HSLB00C; interleukin-6, RD Systems—catalog number D6050).

### 2.7. Data Management

Each patient allocated in the study was given a unique code generated by the independent researcher who created the randomization list. The codes were stored in a password-protected file that was not accessible to the data management team or other investigators. All data (outcomes, questionnaires, clinical data, CT, and laboratory measurements) were retrieved from the online official medical records of the hospital and were immediately uploaded into REDCap (https://redcap.vanderbilt.edu (accessed on 20 April 2020)). Data were retrieved daily by one investigator using a datasheet available online and in print form. Collected data were sent by email to two researchers on the data management team in order to establish double data entry.

### 2.8. Outcomes

The main objective of the study was to determine safety for the use of icatibant and iC1e/K in severe COVID-19 patients. We also evaluated time to clinical improvement (TTCI) as defined by the Cap-China Network [38], which was employed in the LOTUS China trial (Lopinavir Trial for Suppression of SARS-Cov-2 in China) [11] and recommended by the World Health Organization. TTCI refers to the time from randomization to an improvement of two points on the seven-category ordinal scale as follows [11] (or discharge alive from the hospital): 1, not hospitalized with resumption of normal activities; 2, not hospitalized, but unable to resume normal activities; 3, hospitalized, not requiring supplemental oxygen; 4, hospitalized, requiring supplemental oxygen; 5, hospitalized, requiring nasal high-flow oxygen therapy, non-invasive mechanical ventilation; 6, hospitalized, requiring invasive mechanical ventilation; or 7, death. Additional outcomes were as follows: percentage of patients on seven-category scale on days 7, 14, and 21; time on mechanical ventilation; time of hospitalization of survivors; time on oxygen support; time from randomization to hospital discharge; time from randomization to death; improvement of lung CT-scan scores according to guidelines published elsewhere [37], and frequency of severe adverse events as defined by National Cancer Institute Common Terminology Criteria for Adverse Events (CTCAE), version 5.0. Quick sepsis-related organ failure score (qSOFA) was determined as defined elsewhere (qsofa.org (accessed on 20 April 2020)).

### 2.9. Statistical Analysis

All statistical analyses were performed using SPSS version 22.0 and GraphPad Prism 8.0. Values are presented as means and standard deviation in tables and as means and standard error of the mean in figures. To check the normal distribution of variables, the Kolmogorov–Smirnov test was used. When the variable did not present a normal distribution, the equivalent non-parametric test was used. For comparisons between the three groups at baseline, the unidirectional analysis of variance (ANOVA) was used. For non-parametric variables, the Kruskal–Wallis test was employed. In addition, a paired Student’s *t*-test (or Wilcoxon test) was used to compare variables at admission and at discharge for all three groups. Except for qSOFA and TTCI, all remaining data are presented as admission and discharge, as most patients were discharged about 10–14 days after admission. To test for differences in frequencies between groups, we used the chi-square test. A *p*-value of less than 0.05 was considered statistically significant.

## 3. Results

**Figure 1:**
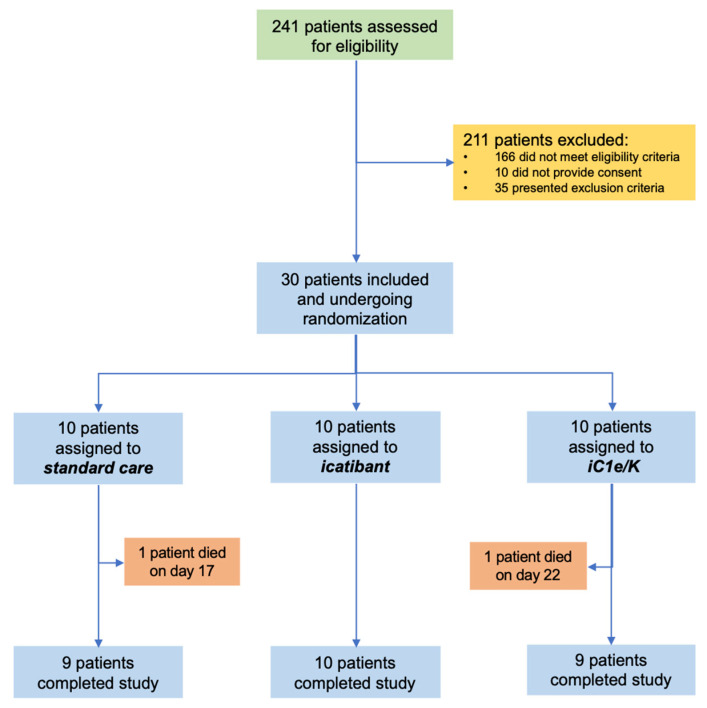
Schematic representation of patient screening, selection, and randomization. iC1e/K, inhibitor of C1 esterase/kallikrein.

From 23 April 2020 to 14 June 2020, 241 patients were assessed for eligibility and 30 were included in the study (Figure 1; Tables S1 and S2). The inclusion criteria were strict in order to assure the inclusion of patients with severe COVID-19 only. The main reasons for exclusions were as follows: (i) despite clinical features compatible with COVID-19, diagnosis was not confirmed by PCR; (ii) beginning of symptoms over 12 days; (iii) baseline O_2_ saturation over 94%. During the study period, the total number of patients screened accounted for 90% of all patients seeking medical attention at the Hospital & Clinics of the University of Campinas due to clinical suspicion of COVID-19. Patients included in the study represented 31% of all patients diagnosed with COVID-19 during this period. Table 1 presents the reasons for the exclusion of screened patients. Table S2 provides the chronological sequence of inclusion and randomization. Ten patients were randomized to each group: standard care, icatibant, and iC1e/K. Throughout the study, supporting medication was provided upon medical decision (Table S3).

Table 1 presents the baseline parameters of all patients included in the study. The mean age was 51 years, 46% were women, and the mean time to hospital admission from the onset of symptoms was eight days. The gender, age, and demographics in each group were similar. The baseline body temperature, blood O_2_ saturation, lung CT score, systolic blood pressure, diastolic blood pressure, white blood cells, blood lymphocytes, blood platelets, plasma glucose, serum creatinine, aspartate aminotransferase (AST), lactate dehydrogenase (LDH), creatine kinase (CK), and C-reactive protein levels in each group were similar (Table 1). At baseline, alanine aminotransferase (ALT) was significantly higher in the icatibant group; nevertheless, all patients in this group had ALT levels within the inclusion range.

Among the 30 patients included in the study, 25 were previously diagnosed with other medical conditions (Table 2). Hypertension was the leading comorbidity, affecting 50% of the patients. Diabetes and obesity were present in 47% and 43% of the patients, respectively. Overweight, dyslipidemia, hypothyroidism, asthma, and fibromyalgia were also present in this cohort. Four patients were former smokers, whereas only one was currently smoking at the time of inclusion in the study (Table 2).

**Figure 2:**
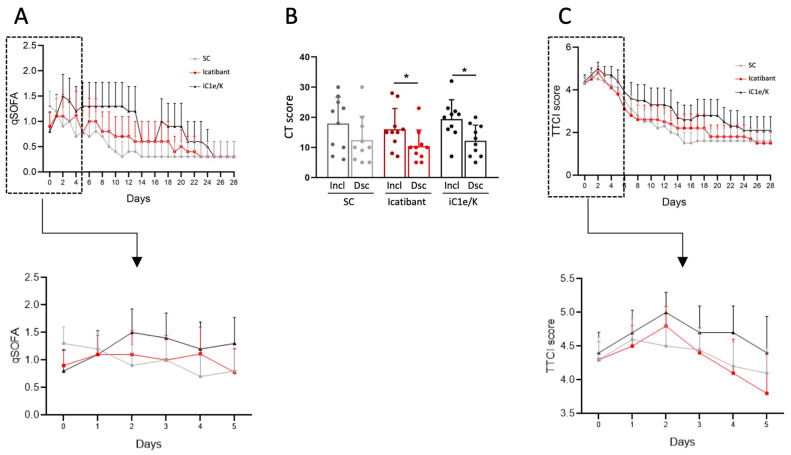
Major clinical and image-related outcomes. The quick sepsis-related organ failure score (qSOFA) was determined daily throughout hospitalization (**A**). Computed tomography lung score was obtained at inclusion and discharge (**B**). Time to clinical improvement (TTCI) was determined daily throughout hospitalization (**C**). Insets represent qSOFA (A) and TTCI (**C**) during the initial five days of treatment. The Kruskal–Wallis test was employed to compare groups (**A**,**C**) and a paired Students *t*-test was used to compare pre- versus post-intervention condition (**B**). Results are presented as mean ± standard deviation. * *p* < 0.05. Dsc, discharge; iC1e/K, inhibitor of C1 esterase/kallikrein; Incl, inclusion; SC, standard care.

Upon inclusion, all patients were diagnosed with severe COVID-19 based on SARS-CoV-2 diagnosis by RT-PCR, low blood O_2_ saturation levels (Table 1), high qSOFA (Figure 2A) and high lung CT score (Table 1; Figure 2B and Figures S1–S3); there were no significant differences in admission severity parameters among the groups.

Neither icatibant nor iC1e/K pharmacological interventions significantly modified mortality and TTCI as compared to the standard care group (Table 3). Overall, there were two deaths, one in the standard care group (on day 17) and one in the iC1e/K group (on day 21). Times from admission to discharge were 10.5, 11.0, and 14.2 days in the standard care, icatibant, and iC1e/K groups, respectively (*p* = 0.62), whereas times in the ICU were 4.6, 6.2, and 8.7 days in the standard care, icatibant, and iC1e/K groups, respectively (*p* = 0.71). Clinical improvement was similar among the groups, as determined by the qSOFA (Figure 2A) and TTCI score (Figure 2C). This was also true when comparison was performed during the initial five days since inclusion in the study, which was the period when pharmacological intervention was undertaken (Figure 2A, inset and Figure 2C, inset). Nevertheless, both, icatibant and iC1e/K promoted significant reductions in lung CT scores (Figure 2B).

**Figure 3:**
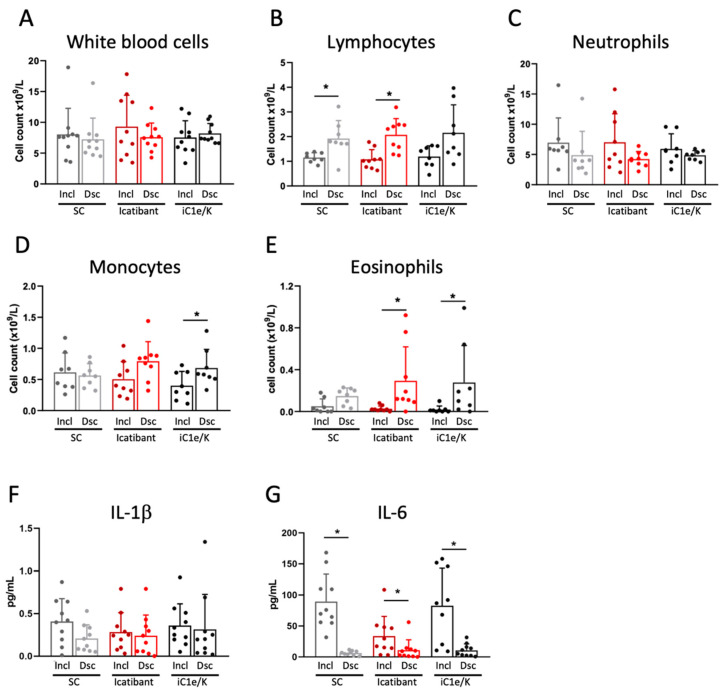
Blood immune cell and cytokine outcomes. White blood cells (**A**), lymphocytes (**B**), neutrophils (**C**), monocytes (**D**), and eosinophils (**E**) were determined by the automated method in blood samples collected at inclusion and discharge. Interleukin-1 beta (**F**) and interleukin-6 were determined using ELISA kits in serum samples collected at inclusion and discharge. Dsc, discharge; iC1e/K, inhibitor of C1 esterase/kallikrein; IL-1b, interleukin-1 beta; IL-6, interleukin-6; Incl, inclusion; SC, standard care. Non-parametric Wilcoxon test was used for comparisons between pre- and post-intervention (**A**–**G**). Results are presented as mean ± standard deviation. * *p* < 0.05.

Neither icatibant nor iC1e/K pharmacological interventions significantly modified blood immune cell counts as compared to the standard care group (Figure 3A–E and Table S4). As compared to inclusion, the discharge white blood cell counts were unchanged in all three groups (Figure 3A, Table S4), lymphocytes were increased in the standard care and icatibant groups (Figure 3B, Table S4), neutrophils were unchanged in all three groups (Figure 3C, Table S4), monocytes were increased in the iC1e/K group (Figure 3D, Table S4), and eosinophils were increased in the icatibant and iC1e/K groups (Figure 3E, Table S4). The intervention did not modify the serum levels of IL-1β (Figure 3F), whereas IL-6 was reduced in all three groups (Figure 3G).

**Figure 4:**
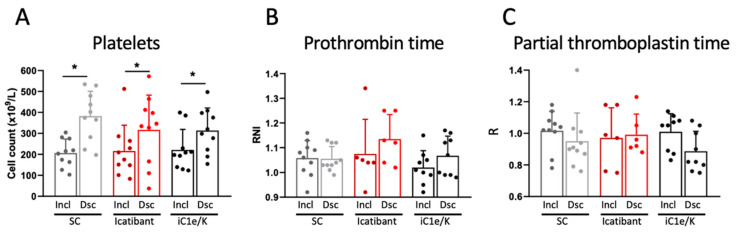
Coagulation-related parameters. Blood platelet counts (**A**), prothrombin time (**B**), and partial thromboplastin time (**C**) were determined in blood samples collected at inclusion and discharge. Dsc, discharge; iC1e/K, inhibitor of C1 esterase/kallikrein; Incl, inclusion; SC, standard care. Non-parametric Wilcoxon test was used for comparisons between pre- and post-intervention. Results are presented as mean ± standard deviation. * *p* < 0.05.

At baseline, parameters related to the coagulation system were similar among all three groups (Table S5). Neither icatibant nor iC1e/K pharmacological interventions significantly modified any of the coagulation parameters evaluated in this study (Figure 4, Table S5). As compared to inclusion, at discharge, blood platelets were increased in all three groups (Figure 4A; Table S5), whereas D-dimer (Table S5), prothrombin time (Figure 4B; Table S5), and partial thromboplastin time (Figure 4C; Table S5) remained unchanged. There were no significant changes in blood levels of urea and creatinine (Table S6).

During the intervention, there were no significant differences in reported adverse effects among the groups (Table S7). As a whole, 14 patients presented with increased AST/ALT, five patients presented with diarrhea, three patients presented with nausea, three patients presented with vomiting, three patients presented with increased blood bilirubin, two patients presented with bradycardia, and none of the patients presented with cardiac arrhythmias. No additional adverse effects were reported in the intervention groups. At discharge, there were no differences in clinical variables among the groups (Table S8).

## 4. Discussion

In this study, we hypothesized that pharmacological inhibition of the kinin–kallikrein system could alleviate the acute inflammatory response during the initial steps of SARS-CoV-2 infection and thus provide a therapeutic advance against COVID-19. We used two drugs currently approved for the treatment of hereditary angioedema, icatibant and iC1e/K, which were compared against standard care alone. As a whole, we could find neither a reduction of mortality nor a reduction of time to clinical improvement. Nevertheless, there were significant reductions of lung CT scores, which could suggest a positive impact of pharmacological intervention on lung recovery, and increases in blood eosinophils, which is regarded as an indicator of disease recovery [38,39]. In addition, interventions promoted no increases in the incidence of adverse effects, suggesting that the drugs are safe for use in patients with severe COVID-19.

Icatibant is an inhibitor of bradykinin receptor 2, which is approved for the treatment of bradykinin-induced edema in hereditary angioedema, leading to rapid relief of symptoms [40]. Side effects are usually restricted to inflammatory signals in the site of injection [41,42]. In hereditary angioedema, a single 30 mg dose of icatibant is capable of reducing symptoms in minute to a few hours [42]. As icatibant has a short half-life (6 h) [43], we used a 30 mg/dose every 8 h for four days, which proved safe, as no increased adverse effects were reported.

iC1e/K is a human-derived protein with an excellent record of beneficial actions in hereditary angioedema [44,45]. It is well tolerated by most patients, and adverse effects are usually restricted to rash and headache [46]. Due to its long half-life (56 h), we used 20 IU/kg at inclusion and on day four. There was no increased appearance of adverse effects in our cohort. It is worthwhile to mention that iC1e/K is present in human plasma, and ongoing clinical studies employing convalescent plasma to treat COVID-19 [47] may benefit not only from the presence of immunoneutralizing antibodies against SARS-CoV-2 but also from its endogenous capacity to inhibit the kinin–kallikrein system, as shown for hereditary angioedema [48,49].

As the comparator, we chose standard care alone, because when the trial began, there was no evidence for a pharmacological approach that could impact positively on COVID-19. Currently, there is at least one study showing a positive impact for dexamethasone reducing mortality in severe COVID-19 [8], and further studies with inhibitors of the kinin–kallikrein system should consider adding dexamethasone to all groups. Placebo and blinding were not adopted because of the urgent nature of this medical condition, and this was approved by the Ethical Review Committee.

The decision to pharmacologically intervene during the first four days after patient enrolment was based on the fact that bradykinin is a fast-acting, acute-phase inflammatory substance that has a very short half-life and is promptly produced upon exposure to the triggering agent [50,51]. We reasoned that if viral interaction could modify ACE2 catalytic activity, bradykinin would accumulate during the initial steps of COVID-19 progression, and pharmacological inhibition would produce clinical outcomes only if used early after infection. Unfortunately, as in all other clinical reports on COVID-19 [2,8,11,12], in this study, the mean time since symptom appearance to hospital admittance was eight days, which could be somewhat late for intervention upon the bradykinin system. This could be one of the reasons explaining why we did not find a reduction in time to clinical improvement in our patients.

Even though interventions were incapable of modifying mortality and time to clinical improvement, the reduction of lung CT scores and increased blood eosinophils could indicate a beneficial impact of intervention on patient recovery. As recently reported, the mean time for poorest lung CT scores, which is defined as peak stage or stage 3, is 9–13 days since the beginning of symptoms [37]. Thus, most patients included in our study were about to enter the time window when stage 3 develops. It is currently unknown if severe COVID-19 acute lung lesions could promote long-term structural abnormalities and, if so, how it could impact on respiratory function. In at least two studies, lung CT abnormalities were shown to persist after four weeks since the beginning of disease [37,52]. Thus, it is intuitive to believe that improved lung CT scores in patients treated with kinin–kallikrein inhibitors indicate lung recovery that could impact on long-term respiratory function. In concert with this hypothesis, a recent study using icatibant to treat a small group of COVID-19 patients reported a reduced need for supplemental oxygen usage following intervention [53]. If future studies show late respiratory loss in patients with COVID-19, it would be interesting to re-evaluate this cohort using functional respiratory tests.

The increased blood eosinophils provide yet another indication of improved recovery in the groups undergoing pharmacological intervention. In fatal cases of COVID-19, blood eosinophil counts were consistently reduced at hospital admittance [54], whereas autopsies revealed an absence of eosinophils in lung specimens [55]. Furthermore, a low blood eosinophil count was identified as a risk factor (odds ratio = 10.2) for severe progression of COVID-19 [56], whereas the progressive increase of blood eosinophils was associated with disease recovery [38]. Recent studies have challenged the classic concept of eosinophil roles being restricted to helminthic parasitosis and allergic diseases [57]. Eosinophils have been shown to act centrally in immunomodulatory networks that warrant homeostasis during inflammatory responses [58] and also to promote antiviral immunity in influenza A virus infection [59]. This has been proposed as the mechanism underlying the protective impact of asthma on influenza morbidity [59], which could also be applied to COVID-19 [60]. Other systemic inflammatory markers measured in this study were not modified by pharmacological intervention. Nevertheless, recent studies have identified early inflammatory markers that indicate severe progression of the disease, and this could be tested in future studies with bradykinin inhibitors. We did not measure blood levels of bradykinin pathway components, because they are very unstable, and systemic levels are not expected to reflect tissue-specific activation of the system. In addition, pharmacological intervention did not modify coagulation markers determined in this study. We acknowledge that the study of earlier and middle-stage physiological and clinical endpoints is an important feature aside from ascertaining safety, and further studies could provide a more specific evaluation of the coagulation system in COVID-19 patients treated with bradykinin inhibitors.

In addition to the bradykinin system, ACE2 interacts also with the renin-angiotensin system and the coagulation system; thus, it has been proposed that SARS-CoV-2/ACE2 interaction could be in the center of the multisystemic abnormalities that are common to patients with severe COVID-19, such as inflammation, coagulopathy, and cardiovascular failure [16]. Bradykinin is an inflammatory peptide produced from high-molecular weight kininogen in a reaction catalyzed by kallikrein [61]. Bradykinin can convey its inflammatory and vasoactive actions through bradykinin receptor 2 or yet be further processed by carboxypeptidase N to produce DR9-bradykinin that acts through bradykinin receptor 1 to deliver pain and inflammatory signals [62]. ACE2 inactivates DR9-bradykinin and, together with angiotensin-converting enzyme (ACE), which inactivates bradykinin, shuts down the kinin–kallikrein system [31]. Angiotensin II is a hormone involved in the regulation of cardiac function, blood pressure, and electrolyte balance [63,64]. It is produced from angiotensin I through the action of ACE, whereas ACE2 catalyzes its degradation, thereby inactivating the renin–angiotensin system [65]. The interaction of ACE2 with the coagulation system occurs via two mechanisms: (i), catalyzing the production of angiotensin 1–9, which reduces plasminogen activator and increases PAI-1, thus inhibiting fibrinolysis [66], and (ii), controlling the activity of kallikrein, which in turn converts plasminogen into plasmin [67]. In our patients, we could not find evidence for an effect of either icatibant or iC1e/K on the regulation of the cardiovascular and coagulation systems. Nevertheless, the effects of both drugs on lung recovery and eosinophil counts suggest that bradykinin could be involved in the inflammatory response to SARS-CoV-2 infection.

The main weaknesses of this study are the small number of patients and the fact that pharmacological intervention was instituted approximately eight days after the beginning of symptoms; both these weaknesses should be taken into consideration when analyzing the positive findings of the study. An important reason for restricting the cohort to 30 patients was that inhibitors of kinin–kallikrein have never been used in COVID-19 patients, and we wanted to test for safety before proposing a larger trial. Regarding the fact that the mean time for patient inclusion was eight days, which could be a late stage for interventions targeting bradykinin, there is little that could be done because of the natural course of the disease, which prompts patients to seek medical attention at this stage. Even if patients were advised to seek medical attention at an earlier stage, most of them would not progress to severe COVID-19. Therefore, only the development of methods that predict severe progression of disease at an early stage could reduce time to inclusion. The additional weaknesses regarding no inclusion of placebo group and no blinding were approved by the Ethical Review Committee because of the urgent nature of investigating new potential approaches to treat COVID-19.

## 5. Conclusions

We report the impact of pharmacological inhibition of the kinin–kallikrein system in patients with severe COVID-19. The lack of findings regarding mortality and time to clinical improvement could be attributed to small sample size, beginning the intervention at a late stage or because bradykinin may not be central to the catalysis of the high mortality of COVID-19. Nevertheless, safety, improvement in CT lung scores, and increased blood eosinophils suggest that intervention had a beneficial impact on patient recovery and should be considered in future trials.

## Figures and Tables

**Table 1:** Baseline parameters of patients included in the study.

Parameter	SC	Icatibant	iC1e/K	*p*	All Patients
**Female/male**	5/5	3/7	6/4	0.39	14/16
**Age (y)**	48.9 ± 10.5	51.6 ± 9.1	54.4 ± 14.8	0.58	51.6 ± 11.5
**BMI (kg/m^2^)**	29.6 ± 7.8	30.2 ± 5.1	32.3 ± 7.0	0.70	30.6 ± 6.7
***BMI >30***	*4*	*4*	*6*	*0.59*	*14 (46%)*
***BMI >25 <30***	*2*	*4*	*1*		*7 (23%)*
**Symptoms onset (d)**	8.8 ± 2.7	7.8 ± 2.2	8.1 ± 2.5	0.57	8.1 ± 2.5
**Body temperature (°C)**	36.3 ± 0.5	36.0 ± 1.2	36.6 ± 0.6	0.41	36.4 ± 1.1
***>37*** **°*C (n)***	*2*	*2*	*2*		*6*
**SatO2 (%)**	92.9 ± 3.3	90.7 ± 2.7	89.8 ± 7.2	0.35	91.4 ± 4.7
**Lung CT score**	17.9 ± 7.8	16.0 ± 6.9	19.4 ± 6.4	0.60	17.8 ± 7.3
**Systolic blood pressure (mmHg)**	117.4 ± 10.7	121.0 ±10.2	127.1 ± 26.0	0.51	124 ± 18
***<90 mmHg (n)***	*0*	*0*	*1*		*1*
**Diastolic blood pressure (mmHg)**	75.5 ± 12.3	79.2 ± 10.4	75.4 ± 15.9	0.91	76.7 ± 12.7
***<60 mmHg (n)***	*0*	*0*	*0*		*0*
**White cell count (×10^9^/L) ¥**	7.78 ± 4.39	7.76 ± 4.98	7.76 ± 2.78	0.99	7.76 ± 4.0
***<4 × 10^9^/L (n)***	*2*	*2*	*1*		*5 (16.7%)*
***4–10 × 10^9^/L (n)***	*7*	*6*	*7*		*20 (66.7%)*
***>10 × 10^9^/L (n)***	*1*	*2*	*2*		*5 (16.7%)*
**Lymphocyte count (×10^9^/L) ¥**	1.08 ± 0.29	1.08 ± 0.39	1.37 ± 0.48	0.45	1.18 ± 0.48
***<1 × 10^9^/L (n)***	*3*	*3*	*3*		*9*
***>1 × 10^9^/L (n)***	*6*	*6*	*6*		*18*
**Platelet count (×10^9^/L) ¥**	217.6 ± 55.8	217.5 ± 123.5	229.9 ± 98.2	0.97	221.7 ± 93.4
***<100 × 10^9^/L (n)***	*0*	*1*	*0*		*1*
*** ≥*** ***100 × 10^9^/L (n)***	*10*	*9*	*10*		*29*
**Plasma glucose (mg/dL) ¥**	176.8 ± 94.9	158.9 ± 91.8	182.4 ± 111.4	0.81	172.7 ± 96.2
***<125 (mg/dL) (n)***	*4*	*6*	*3*		*13*
***>125 (mg/dL) (n)***	*6*	*4*	*7*		*17*
**Serum creatinine (mg/dL) ¥**	0.9 ± 0.3	1.2 ± 0.9	0.9 ± 0.3	0.34	1.00 ± 0.61
***<1.2 (mg/dL) (n)***	*9*	*8*	*9*		*26*
***>1.2 (mg/dL) (n)***	*1*	*2*	*1*		*4*
**AST (U/L) ¥**	63.0 ± 54.0	82.7 ± 88.9	37.3 ± 12.7	0.13	61.8 ± 62.2
***<40 (U/L) (n)***	*4*	*3*	*4*		*11*
***>40 (U/L) (n)***	*6*	*7*	*5*		*18*
**ALT (U/L) ¥**	38.6 ± 35	76.7 ± 76.4	27.6 ± 13	<0.01	48.3 ± 52.7
***<40 (U/L) (n)***	*7*	*2*	*8*		*17*
***≥*** ***40 (U/L) (n)***	*3*	*8*	*1*		*12*
**LDH (U/L) ¥**	308.7 ± 55.4	396.5 ± 135.2	376.5 ± 254.7	0.91	362.2 ± 176.0
***<245 (U/L) (n)***	*1*	*2*	*2*		*5*
***≥*** ***245 (U/L) (n)***	*7*	*8*	*8*		*23*
**CK (U/L) ¥**	282.7 ± 245.8	299.0 ± 359.2	123.0 ± 99.9	0.24	221.0 ± 213.5
***<185 (U/L) (n)***	*4*	*1*	*5*		*10*
***≥*** ***185 (U/L) (n)***	*3*	*1*	*1*		*4*
**C-Reactive Protein (mg/L) ¥**	123.2 ± 61.8	109.0 ± 81.4	110.9 ± 86.3	0.76	117.6 ± 74.3
***<10 (mg/L) (n)***	*10*	*10*	*9*		*29*
***≥*** ***10 (mg/L) (n)***	*0*	*0*	*0*		*0*

Abbreviations: ALT, alanine aminotransferase; AST, aspartate aminotransferase; BMI, body mass index; CK, creatine kinase; iC1e/K, inhibitor of C1 esterase/kallikrein; LDH, lactate dehydrogenase; SC, standard care. One-way ANOVA/¥ non-parametric distribution (Kruskall–Wallis), chi-square test (for all variables in which the expected numbers were too small no significance was calculated).

**Table 2:** Pre-morbid conditions of patients included in the study.

Comorbidities at baseline	All patients (n:30)	SC (n:10)	Icatibant (n:10)	iC1e/K (n:10)	*p*-Value
Hypertension, n (%)	15 (50)	4 (40)	4 (40)	7 (70)	0.30
Diabetes Mellitus, n (%)	14 (46.7)	5 (50)	4 (40)	5 (50)	0.88
Obesity, n (%)	13 (43.3)	3 (30)	4 (40)	6 (60)	0.39
Overweight, n (%)	7 (23.3)	2 (20)	4 (40)	1 (10)	--
Dyslipidemia, n (%)	5 (16.67)	1 (10)	1 (10)	3 (30)	--
Former smoker, n (%)	4 (13.3)	1 (10)	1 (10)	2 (20)	--
Hypothyroidism, n (%)	2 (6.67)	0 (0)	0 (0)	2 (20)	--
Asthma, n (%)	1 (3.33)	0 (0)	0 (0)	1 (10)	--
Current smoker, n (%)	1 (3.33)	1 (10)	0 (0)	0 (0)	--
Fibromyalgia, n (%)	1 (3.33)	0 (0)	1 (10)	0 (0)	--

Abbreviations: iC1e/K, inhibitor of C1 esterase/kallikrein; SC, standard care. Chi-square test (for all variables in which the expected numbers were too small no significance was calculated).

**Table 3:** Primary outcomes of patients included in the study.

	SC	Icatibant	iC1e/K	*p*	All Patients
TATD (days)	10.5 ± 7.1	11.0 ± 8.9	14.2 ± 10.1	0.62	11.9 ± 8.6
TIICU (days)	4.6 ± 8.9	6.2 ± 10.5	8.7 ± 11.8	0.71	6.5 ± 10.2
Deaths	1	0	1		2

Abbreviations: iC1e/K, inhibitor of C1 esterase/kallikrein; TATD, time from admission to discharge; TIICU, time in intensive care unit. One-way ANOVA/chi-square test (for all the variables in which the expected numbers that were too small no significance was calculated).

## Data Availability

All data obtained in this study can be made available to qualified researchers upon request, provided that data protection and ethical standards are in compliance with the principles of the Declaration of Helsinki.

## Supplementary Materials

The following are available at https://www.mdpi.com/1999-4915/13/2/309/s1, Table S1: Patients screened and excluded from study; Table S2: Patients included in the study; Table S3: Supporting medication; Table S4: Blood cell counts; Table S5: Blood coagulation parameter values; Table S6: Parameters related to renal function; Table S7: Adverse effects during intervention; Table S8: Clinical variables at discharge; Supplementary Figure S1: Computed tomography lung scans obtained from patients randomized to standard care; Supplementary Figure S2: Computed tomography lung scans obtained from patients randomized to icatibant; Supplementary Figure S3: Computed tomography lung scans obtained from patients randomized to inhibitor of C1 esterase/kallikrein (iC1e/K).

## Abbreviations

ACE2	Angiotensin-converting enzyme 2
ALT	Alanine aminotransferase
ANOVA	Analysis of variance
AST	Aspartate aminotransferase
CK	Creatine kinase
COVID-19	Coronavirus disease 19
CT	Computed tomography
CTCAE	National Cancer Institute Common Terminology Criteria for Adverse Events
ELISA	Enzyme-linked immunosorbent assay
HCG	Human chorionic gonadotrophin
HIV	Human immunodeficiency virus
iC1e/K	Inhibitor of C1 esterase/kallikrein
ICU	Intensive care unit
LDH	Lactate dehydrogenase
qSOFA	Quick sepsis-related organ failure score
RT-PCR	Real-time polymerase chain reaction
RTC	Randomized controlled trial
TTCI	Time to clinical improvement
