# Supplementary material for: Safety and Outcomes Associated with the Pharmacological Inhibition of the Kinin–Kallikrein System in Severe COVID-19
Source: Viruses. 2021 Feb 16;13(2):309. doi: 10.3390/v13020309 (PMC7920028; doi:10.3390/v13020309)

Supplementary data

Safety and outcomes associated with the pharmacological inhibition of the kinin-kallikrein system in severe COVID-19

Eli Mansour[1], Andre C. Palma[1], Raisa G. Ulaf[1], Luciana C. Ribeiro[1], Ana Flavia Bernardes[1], Thyago A. Nunes[1], Marcus V. Agrela[1], Bruna Bombassaro[2], Milena Monfort-Pires[2], Rafael L. Camargo[2], Eliana P. Araujo[2,3], Natalia S. Brunetti[4], Alessandro S. Farias[4], Antônio Luís E. Falcão[5], Thiago Martins Santos[1], Plinio Trabasso[1], Rachel P. Dertkigil[6], Sergio S. Dertkigil[6], Maria Luiza Moretti[1], Licio A. Velloso[1,2]

Supplementary data

Supplementary Table 1. Patients screened and excluded from study.

Excluded Patients	Age	Gender	Exclusion Criteria
**E1**	55	M	Symptoms >12 days
**E2**	63	F	Symptoms >12 days
**E3**	58	M	Symptoms >12 days
**E4**	43	M	Symptoms >12 days
**E5**	71	F	Symptoms >12 days
**E6**	56	M	Symptoms >12 days
**E7**	75	F	Symptoms >12 days
**E8**	48	M	Symptoms >12 days
**E9**	65	F	Symptoms >12 days
**E10**	55	M	Symptoms >12 days
**E11**	68	M	Symptoms >12 days
**E12**	71	F	Symptoms >12 days
**E13**	59	F	Symptoms >12 days
**E14**	40	M	Sat >94%
**E15**	42	M	Sat >94%
**E16**	41	M	Sat >94%
**E17**	25	M	Sat >94%
**E18**	56	M	Sat >94%
**E19**	32	F	Sat >94%
**E20**	66	F	Sat >94%
**E21**	31	M	Sat >94%
**E22**	37	M	Kidney transplant
**E23**	57	M	Kidney transplant
**E24**	51	M	Kidney transplant
**E25**	35	F	Kidney transplant
**E26**	19	F	Kidney transplant
**E27**	42	F	Kidney transplant
**E28**	96	M	DNPC
**E29**	82	M	DNPC
**E30**	94	M	DNPC
**E31**	85	M	DNPC
**E32**	82	F	DNPC
**E33**	59	M	DNPC

**E34**	23	M	DNPC
**E35**	63	M	DNPC
**E36**	52	M	DNPC
**E37**	64	M	DNPC
**E38**	66	M	CKD
**E39**	54	F	CKD
**E40**	67	F	CKD
**E41**	52	M	CKD
**E42**	59	F	HIV
**E43**	24	M	HIV
**E44**	58	F	HIV
**E45**	69	M	AMI
**E46**	70	M	AMI
**E47**	51	F	AMI
**E48**	54	F	AMI
**E49**	54	M	Hydroxycloroquine use
**E50**	59	F	Hydroxycloroquine use
**E51**	43	M	Hydroxycloroquine use
**E52**	27	M	*Systemic sclerosis*
**E53**	62	F	*Systemic sclerosis*
**E54**	67	M	Stroke
**E55**	88	M	Stroke
**E56**	42	M	Thromboembolic event
**E57**	61	M	Pulmonary thromboembolism
**E58**	59	M	Hepatic cirrhosis
**E59**	40	M	Liver transplant
**E60**	45	F	Autoimmune hepatitis
**E61**	57	M	AML
**E62**	48	M	ALL
**E63**	63	M	Miastenia gravis
**E64**	57	M	FSGS
**E65**	28	M	Polytrauma
**E66**	65	F	Expansive brain injury
**E67**	70	F	ND
**E68**	32	F	ND
**E69**	69	F	ND
**E70**	21	M	ND
**E71**	73	F	ND
**E72**	49	M	ND
**E73**	53	F	ND
**E74**	84	M	ND
**E75**	67	F	ND
**E76**	51	M	ND
**E77**	54	F	ND
**E78**	50	F	ND
**E79**	60	F	ND
**E80**	32	M	ND
**E81**	69	F	ND
**E82**	69	F	ND
**E83**	52	F	ND
**E84**	36	F	ND
**E85**	18	F	ND
**E86**	76	F	ND
**E87**	38	M	ND
**E88**	52	F	ND
**E89**	21	M	ND
**E90**	71	M	ND
**E91**	67	M	ND
**E92**	95	F	ND
**E93**	54	F	ND
**E94**	56	F	ND
**E95**	43	F	ND
**E96**	25	M	ND
**E97**	50	M	ND
**E98**	68	M	ND
**E99**	43	M	ND
**E100**	84	M	ND

**E101**	22	M	ND
**E102**	56	M	ND
**E103**	59	M	ND
**E104**	95	M	ND
**E105**	77	F	ND
**E106**	21	F	ND
**E107**	65	M	ND
**E108**	51	M	ND
**E109**	68	F	ND
**E110**	32	M	ND
**E111**	74	F	ND
**E112**	75	M	ND
**E113**	86	M	ND
**E114**	76	M	ND
**E115**	64	M	ND
**E116**	62	M	ND
**E117**	32	F	ND
**E118**	80	F	ND
**E119**	56	M	ND
**E120**	36	F	ND
**E121**	49	F	ND
**E122**	62	M	ND
**E123**	38	M	ND
**E124**	75	M	ND
**E125**	67	F	ND
**E126**	65	M	ND
**E127**	66	F	ND
**E128**	55	M	ND
**E129**	25	F	ND
**E130**	73	M	ND
**E131**	75	F	ND
**E132**	27	M	ND
**E133**	31	M	ND
**E134**	53	F	ND
**E135**	74	F	ND
**E136**	37	M	ND
**E137**	66	M	ND
**E138**	25	M	ND
**E139**	79	M	ND
**E140**	40	M	ND
**E141**	40	M	ND
**E142**	53	M	ND
**E143**	80	M	ND
**E144**	67	M	ND
**E145**	81	M	ND
**E146**	32	F	ND
**E147**	62	M	ND
**E148**	76	M	ND
**E149**	60	M	ND
**E150**	58	F	ND
**E151**	46	F	ND
**E152**	42	M	ND
**E153**	63	M	ND
**E154**	54	M	ND
**E155**	72	M	ND
**E156**	75	F	ND
**E157**	64	M	ND
**E158**	55	M	ND
**E159**	59	F	ND
**E160**	53	M	ND
**E161**	66	M	ND
**E162**	59	M	ND
**E163**	50	M	ND
**E164**	65	M	ND
**E165**	65	M	ND
**E166**	60	M	ND
**E167**	26	M	ND

**E168**	39	F	ND
**E169**	87	F	ND
**E170**	60	M	ND
**E171**	70	M	ND
**E172**	68	M	ND
**E173**	68	M	ND
**E174**	71	F	ND
**E175**	52	M	ND
**E176**	80	F	ND
**E177**	72	F	ND
**E178**	81	M	ND
**E179**	53	M	ND
**E180**	48	F	ND
**E181**	58	F	ND
**E182**	51	M	ND
**E183**	49	F	ND
**E184**	46	M	ND
**E185**	50	M	ND
**E186**	55	M	ND
**E187**	80	F	ND
**E188**	83	F	ND
**E189**	69	M	ND
**E190**	74	M	ND
**E191**	79	M	ND
**E192**	31	F	ND
**E193**	47	M	ND
**E194**	51	F	ND
**E195**	59	M	ND
**E196**	62	M	ND
**E197**	70	M	ND
**E198**	52	F	ND
**E199**	59	F	ND
**E200**	72	F	ND
**E201**	74	F	ND
**E202**	59	F	ND
**E203**	75	M	ND
**E204**	32	M	ND
**E205**	53	M	ND
**E206**	75	F	ND
**E207**	45	M	ND
**E208**	72	F	ND
**E209**	28	M	ND
**E210**	60	F	ND
**E211**	38	F	ND

Abbreviations: ALL, acute lymphoid leukemia; AMI, acute myocardial infarction; AML, acute myeloid leukemia; CKD, chronic kidney disease; DNPC, did not provide consent; FSGS, focal segmental glomerulosclerosis; HIV, diagnosis of HIV infection; ND, no confirmation of diagnosis by PCR.

Supplementary Table 2. Patients included in the study.

Patient Identity	Age	Gender	Inclusion date	Time since beginning of symptoms (days)	Randomization	Arm
**P1**	50	M	2020-04-23	7	001 YD6	SC
**P2**	57	M	2020-04-24	8	002 ZE3	Icatibant
**P3**	74	F	2020-04-29	4	003 510	iC1e/K
**P4**	30	M	2020-05-01	10	004 SU3	SC
**P5**	47	M	2020-05-01	8	005 DH1	Icatibant
**P6**	57	M	2020-05-04	9	006 GK4	Icatibant
**P7**	47	M	2020-05-07	10	007 OH1	SC
**P8**	47	M	2020-05-14	6	008 IE5	iC1e/K
**P9**	38	M	2020-05-15	4	009 ZL0	Icatibant
**P10**	53	M	2020-05-19	11	010 UW5	Icatibant
**P11**	62	F	2020-05-19	7	011 YB8	iC1e/K
**P12**	57	M	2020-05-20	10	012 UU6	iC1e/K
**P13**	63	F	2020-05-23	7	013 DU2	SC
**P14**	50	M	2020-05-26	12	014 KL6	SC
**P15**	49	F	2020-05-26	10	015 GS8	SC
**P16**	43	M	2020-05-27	10	016 TL8	Icatibant
**P17**	54	F	2020-05-27	7	017 TT4	SC
**P18**	46	F	2020-05-27	11	018 DC5	iC1e/K
**P19**	34	F	2020-05-27	11	019 MI3	SC
**P20**	65	M	2020-05-28	8	020 VZ9	iC1e/K
**P21**	40	M	2020-05-29	5	021 QR4	Icatibant
**P22**	38	F	2020-06-02	3	022 FM6	iC1e/K
**P23**	43	F	2020-06-04	7	023 WG1	SC
**P24**	36	M	2020-06-04	10	024 CC2	iC1e/K
**P25**	42	F	2020-06-08	8	025 VK0	iC1e/K
**P26**	65	M	2020-06-08	5	026 RL1	SC
**P27**	77	F	2020-06-10	10	027 ZR9	iC1e/K
**P28**	66	F	2020-06-12	9	028 LQ8	Icatibant
**P29**	57	F	2020-06-14	8	029 GG3	Icatibant
**P30**	58	F	2020-06-14	6	030 QT1	Icatibant

Abbreviations: iC1e/K, inhibitor of C1 esterase/kallikrein; SC, standard care.

Supplementary Table 3. Supporting medication.

Patient	Group	Supporting Medication
**P1**	1	None
**P2**	2	Dipyrone
**P3**	3	Dipyrone/Clavulanic acid/Azithromycin/Heparin/Insulin/Vasoactive drugs
**P4**	1	None
**P5**	2	Dipyrone/Azithromycin
**P6**	2	Dipyrone
**P7**	1	Dipyrone/Azithromycin/Metformin
**P8**	3	Dipyrone/Azithromycin
**P9**	2	None
**P10**	2	Losartan
**P11**	3	Losartan
**P12**	3	Dipyrone/Azithromycin/Insulin/Losartan
**P13**	1	Dipyrone
**P14**	1	Dipyrone/Clavulanic acid/Amoxicillin/Captopril
**P15**	1	Insulin Dipyrone/Clavulanic acid/Azithromycin/Heparin/Vasoactive drugs
**P16**	2	Dipyrone/Clavulanic acid/Azithromycin
**P17**	1	Hydrochlorothiazide/Losartan
**P18**	3	Dipyrone/Clavulanic acid/Azithromycin/Insulin
**P19**	1	Insulin
**P20**	3	Dipyrone
**P21**	2	None
**P22**	3	Dipyrone/Clavulanic acid/Azithromycin
**P23**	1	Amoxicillin
**P24**	3	Dipyrone/Clavulanic acid/Azithromycin/Heparin
**P25**	3	None
**P26**	1	Dipyrone/Losartan/L-thyroxin/ Clavulanic acid/Azithromycin
**P27**	3	None
**P28**	2	Enalapril/Metformin/Insulin
**P29**	2	Enalapril/Metformin/Insulin
**P30**	2	Metformin/Insulin

Group 1, standard care; group 2, icatibant; group 3, inhibitor of C1 esterase/kallikrein

Supplementary Table 4. Blood cell counts.

	Standard Care			Icatibant			iC1e/K			Total sample		
	Admission	Discharge	p	Admission	Discharge	p	Admission	Discharge	p	Admission	Discharge	p
**White blood cells (x10**3/µL)¥**	7.78 ± 4.38	7.27 ± 3.49	0.96	7.76 ± 4.98	7.87 ± 2.37	0.88	7.76 ± 2.76	7.79 ± 1.65	0.96	7.76 ± 4.00	7.64 ± 2.53	0.88
**Segmented neutrophils (x10**3/µL)¥**	5.81 ± 4.74	4.74 ± 4.00	0.16	6.60 ± 3.61	4.24 ± 1.24	0.05	5.80 ± 2.18	5.0 ± 0.82	0.85	6.08 ± 3.03	4.66 ± 2.30	0.03
**Lymphocytes (x10**3/µL)¥**	1.15 ± 0.20	1.92 ± 0.73	0.02	1.08 ± 0.39	2.07±0.66	0.01	1.19 ± 0.43	2.16 ± 1.13	0.05	1.14 ± 0.35	2.05 ± 0.83	0.01
**Monocytes (x10**3/µL)¥**	0.61 ± 0.31	0.57± 0.19	0.62	0.50 ± 0.28	0.79 ± 0.31	0.07	0.40 ± 0.23	0.69± 0.30	0.03	0.51 ± 0.28	0.69 ± 0.28	0.03
**Eosinophils (x10**3/µL)¥**	0.05 ± 0.07	0.15 ± 0.08	0.08	0.02 ± 0.07	0.15 ± 0.08	0.01	0.02 ± 0.03	0.28 ± 0.35	0.02	0.03 ± 0.05	0.24 ± 0.28	0.01

Abbreviation: iC1e/K, inhibitor of C1 esterase/kallikrein; Paired t test or non-parametric equivalent used for comparisons between pre- and post-intervention. ¥ Non-parametric test.

Supplementary Table 5. Blood coagulation parameter values.

	Standard care			Icatibant			iC1e/K			Total sample		
	Admission	Discharge	p	Admission	Discharge	p	Admission	Discharge	p	Admission	Discharge	p
D-dimer¥	1070.8 ± 745.5	2345.0 ± 3928.9	0.26	834.4 ± 398.2	2429.6 ± 3181.0	0.14	8984.5 ± 24961.8	2753.4 ± 4085.7	0.72	3821.1 ± 14,942	2518.4 ± 3629.0	0.17
Platelets (x10**3/µL) ¥	206.3 ± 66.8	382.0 ± 119.0	<0.01	215.8 ± 123.2	316.5 ± 165.8	0.02	221.4 ± 97.21	313.7 ± 107.64	<0.01	214.5 ± 95.2	337.4 ± 132	<0.01
PT (sec) ¥	12.51 ± 0.86	12.36 ± 0.49	0.57	12.81 ± 1.73	13.40 ± 1.38	0.75	12.06 ± 0.83	12.6 ± 0.97	0.31	12.42 ± 1.11	12.69 ± 0.99	0.55
PT (%)¥	90.75 ± 12.88	90.87 ± 7.85	0.88	90.18 ± 18.26	80.25 ± 12.38	0.46	97.49 ± 12.57	89.37 ± 12.52	0.21	93.04 ± 14.00	87.78 ± 11.24	0.28
PT (RNI) ¥	1.06 ± 0.07	1.06 ± 0.05	0.92	1.07 ± 0.14	1.14 ± 0.10	0.50	1.02 ± 0.07	1.06 ± 0.08	0.21	1.05 ± 0.09	1.08 ± 0.08	0.27
PTT (sec) ¥	29.23 ± 3.57	27.39 ± 5.15	0.31	27.61 ± 5.08	28.03 ± 3.70	0.74	29.09 ± 3.28	25.56 ± 3.65	0.07	28.75 ±3.83	26.93 ± 4.27	0.07
PTT - R¥	1.02 ± 0.12	0.95 ± 0.18	0.31	0.97 ± 0.19	0.99 ± 0.13	0.91	1.01 ± 0.11	0.89 ± 0.13	0.07	1.00 ± 0.13	0.94 ± 0.15	0.07

Abbreviations: iC1e/K, inhibitor of C1 esterase/kallikrein; PT, prothrombin time; PTT, partial thromboplastin time. Paired t test or non-parametric equivalent used for comparisons between pre- and post-intervention. ¥Non-parametric test.

Supplementary Table 6. Parameters related to renal function.

	Standard care			Icatibant			iC1e/K			Total sample		
	Admission	Discharge	p	Admission	Discharge	p	Admission	Discharge	p	Admission	Discharge	p
**Creatinine (mg/dL)** ¥	0.9 ± 0.3	1.0 ± 1.0	0.44	1.2 ± 1.0	1.6 ± 2.3	0.51	0.9 ± 0.3	0.8 ± 0.4	0.39	1.0 ± 0.6	1.1 ±1.5	0.21
**Urea (mg/dL)** ¥	37.6 ± 20.0	38.4 ± 33.5	0.94	40.6 ± 29.1	38.6 ± 25.4	0.62	31.4 ± 10.3	39.1 ± 38.6	0.96	34.5 ± 20.9	38.7 ± 31.7	0.68

Abbreviation: iC1e/K, inhibitor of C1 esterase/kallikrein. Paired t test or non-parametric equivalent used for comparisons between pre- and post-intervention. ¥Non-parametric test.

Supplementary Table 7. Adverse effects during intervention.

Adverse Effect	Total	Standard care	Icatibant	iC1e/K	P
**Increased ASLT/ALT, n (%)**	14 (46.6)	4 (40)	5 (50)	5 (50)	0.88
**Diarrhea n (%)**	5 (16.6)	1 (10)	2 (20)	2 (20)	--
**Nausea, n (%)**	3 (10)	1 (10)	0 (0)	2 (20)	--
**Vomits, n (%)**	3 (10)	1 (10)	1 (10)	1 (10)	--
**Hyperbilirubinemia, n (%)**	3 (10)	2 (20)	1 (10)	0 (0)	--
**Bradycardia, n (%)**	2 (6.6)	1 (10)	0 (0)	1 (10)	--
**Arrhythmia, n (%)**	0 (0)	0 (0)	0 (0)	0 (0)	--

Abbreviation: iC1e/K, inhibitor of C1 esterase/kallikrein. Chi-square test (for all variables in which the expected numbers were too small no significance was calculated).

Supplementary Table 8. Clinical variables at discharge

	Standard care	Icatibant	iC1e/K	p between groups
**Lung CT score**	**10.44 ± 5.22**	**10.4 ± 5.34**	**13.4 ± 7.89**	0.49
**White Blood Cells (x10**3/µL) ¥**	**7.38 ± 3.41**	**7.72 ± 2.18**	**8.29 ± 2.14**	0.74
**Limphocytes (x10**3/µL) ¥**	**1.71 ± 0.71**	**1.85 ± 0.62**	**2.2 ± 1.03**	0.49
**Segmented neutrophils (x10**3/µL) ¥**	**5.31 ± 3.78**	**4.46 ± 1.46**	**5.39 ± 1.91**	0.74
**Monocytes (x10**3/µL)**	**0.55 ± 0.19**	**0.68 ± 0.24**	**0.62 ± 0.32**	0.58
**Eosinophils (x10**3/µL) ¥**	**0.12 ± 0.08**	**0.23 ± 0.25**	**0.28 ± 0.31**	0.40
**IL-1B (pg/mL) ¥**	**0.21 ± 0.16**	**0.24 ± 0.24**	**0.32 ± 0.41**	0.67
**IL-6 (pg/mL) ¥**	**6.38 ± 3.56**	**10.98 ± 16.69**	**10.83 ± 10.2**	0.63
**Platelets (pg/mL¥)**	**382 ± 118.99**	**316.5 ± 165.78**	**313.7 ± 107.64**	0.44
**Prothrombine time (RNI) ¥**	**1.06 ± 0.05**	**1.13 ± 0.09**	**1.07 ± 0.08**	0.10
**Partial thromboplastine time (R) ¥**	**0.95 ± 0.18**	**0.97 ± 0.12**	**0.89 ± 0.13**	0.49

One-way ANOVA or non-parametric equivalent test was used to compare groups. ¥Non-parametric test.

Supplementary Figure 1. Computed tomography lung scans obtained from patients randomized to standard care. Representative images were selected from the most affected region of the lungs. Scans were obtained at admission and discharge. Patient identification refers to randomization sequence as defined in Supplementary Table 2. Patient #15 missing discharge image is due to death. Each of the five lung lobes was scored 0-5 according to the following parameters: 0, no involvement; 1, <5% involvement; 2, 5-25% involvement; 3, 26-49% involvement; 4, 50-75% involvement; 5, >75% involvement.

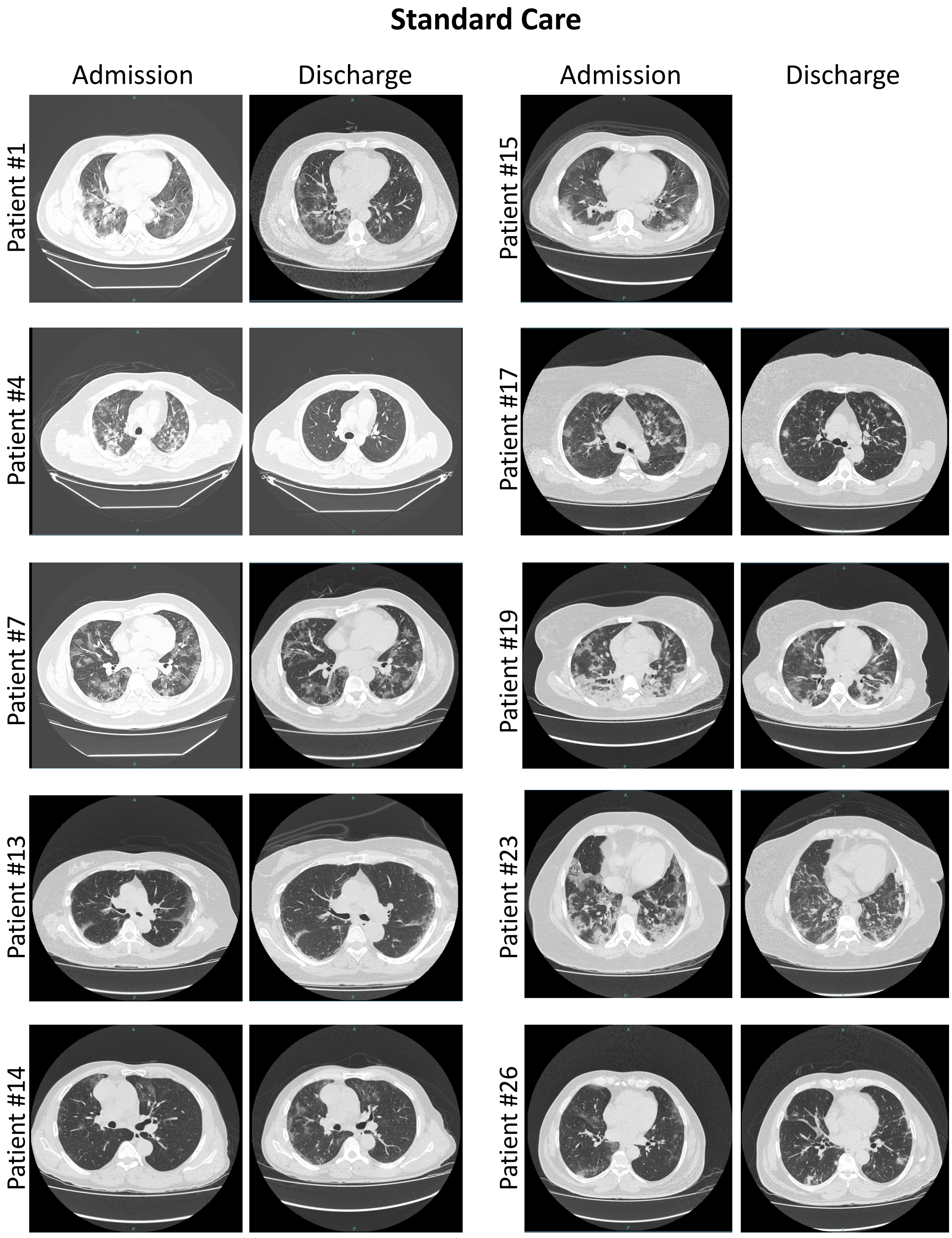

Supplementary Figure 2. Computed tomography lung scans obtained from patients randomized to icatibant. Representative images were selected from the most affected region of the lungs. Scans were obtained at admission and discharge. Patient identification refers to randomization sequence as defined in Supplementary Table 2. Each of the five lung lobes was scored 0-5 according to the following parameters: 0, no involvement; 1, <5% involvement; 2, 5-25% involvement; 3, 26-49% involvement; 4, 50-75% involvement; 5, >75% involvement.

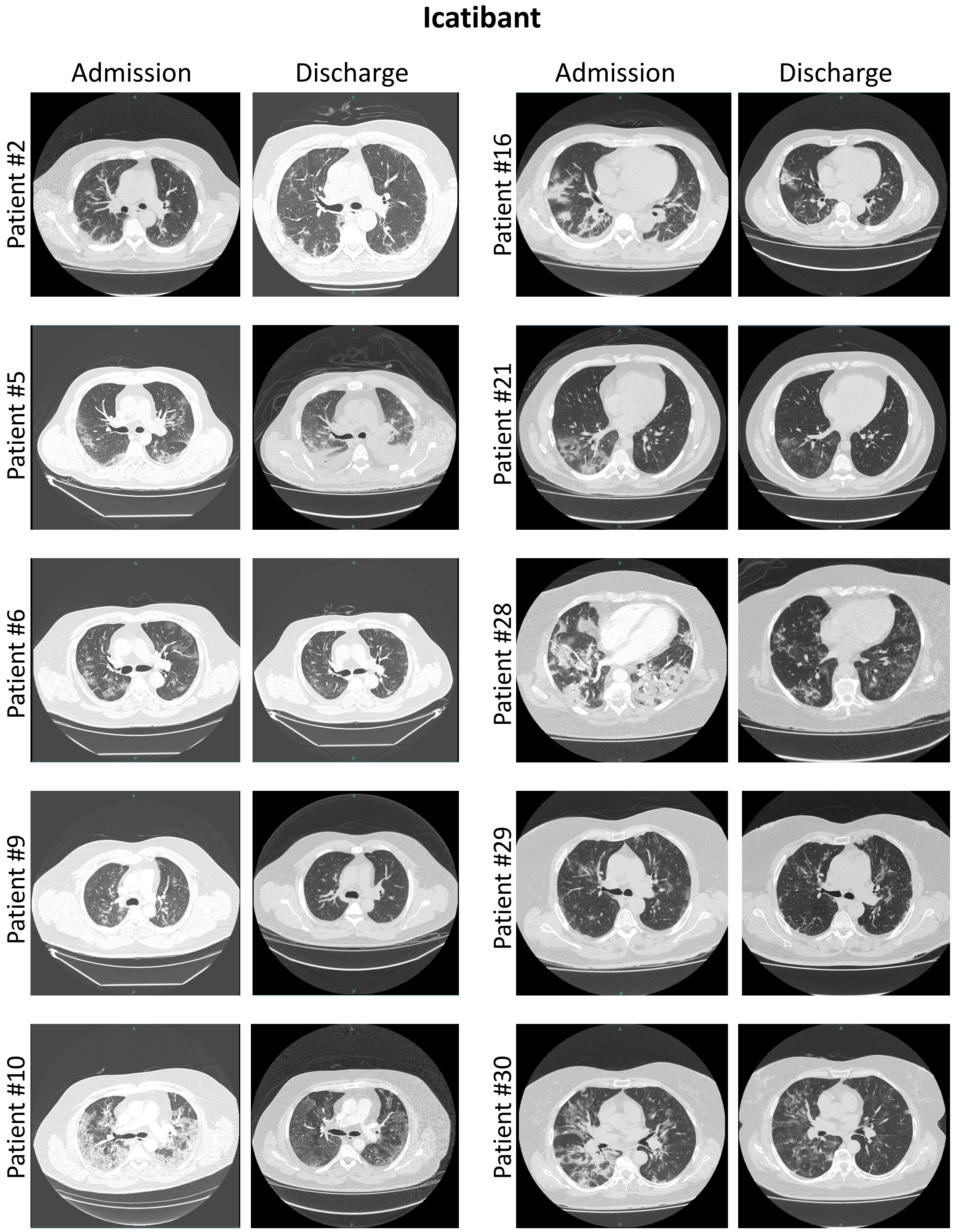

Supplementary Figure 3. Computed tomography lung scans obtained from patients randomized to inhibitor of C1 esterase/kallikrein (iC1e/K). Representative images were selected from the most affected region of the lungs. Scans were obtained at admission and discharge. Patient identification refers to randomization sequence as defined in Supplementary Table 2. Patient #3 missing discharge image is due to death. Each of the five lung lobes was scored 0-5 according to the following parameters: 0, no involvement; 1, <5% involvement; 2, 5-25% involvement; 3, 26-49% involvement; 4, 50-75% involvement; 5, >75% involvement.

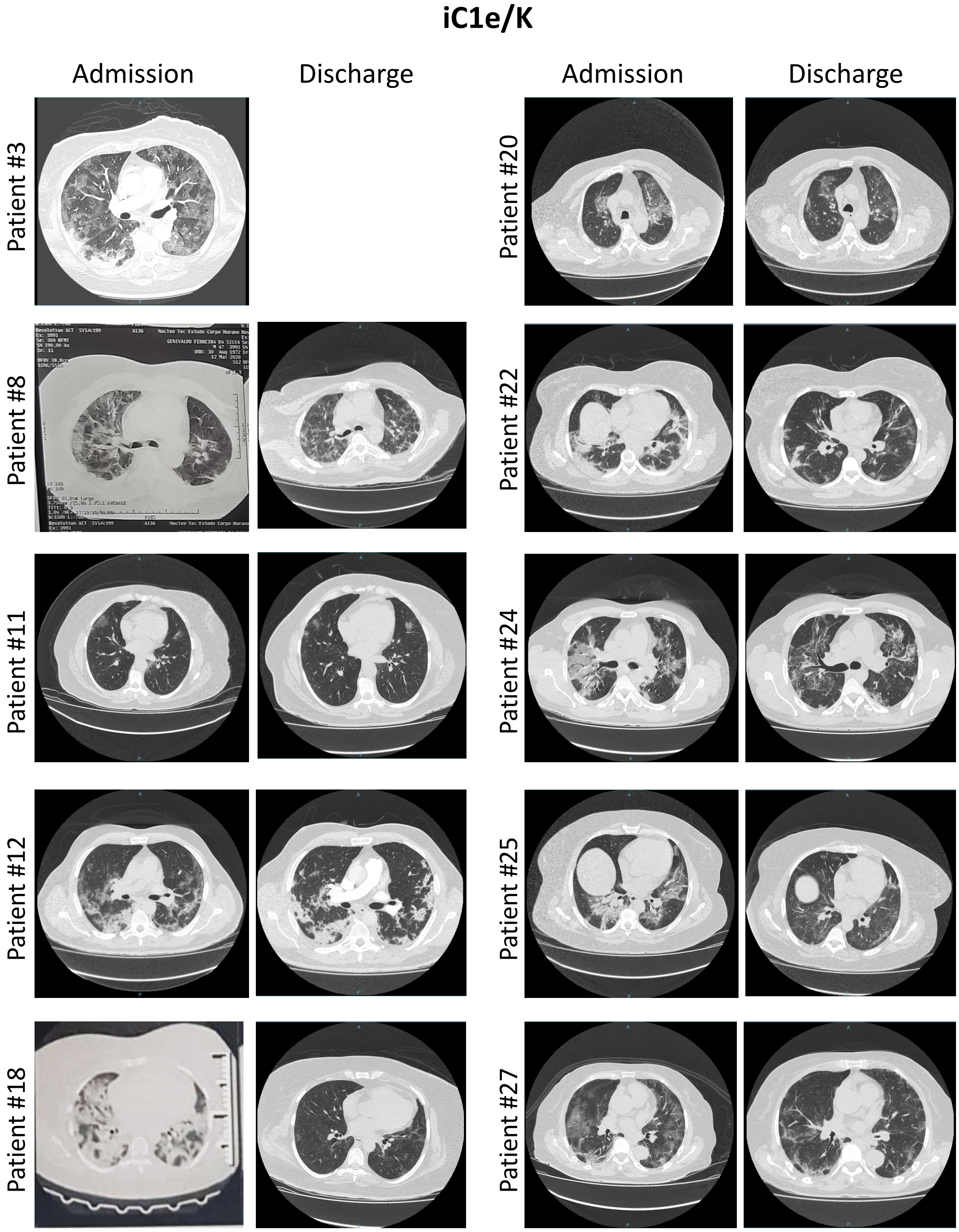

Supplement: Supplementary file 1 [file viruses-13-00309-s001.pdf]
